# The effect of electroconvulsive therapy on neuroinflammation, behavior and amyloid plaques in the 5xFAD mouse model of Alzheimer’s disease

**DOI:** 10.1038/s41598-021-83998-0

**Published:** 2021-03-01

**Authors:** Martina Svensson, Gustaf Olsson, Yiyi Yang, Sara Bachiller, Maria Ekemohn, Joakim Ekstrand, Tomas Deierborg

**Affiliations:** 1 Experimental Neuroinflammation Laboratory, Department of Experimental Medical Sciences, Lund University, Lund, Sweden; 2 Psychiatric Neuromodulation Unit, Department of Clinical Sciences, Lund University, Lund, Sweden

**Keywords:** Alzheimer's disease, Depression, Neuroimmunology

## Abstract

Microglial cells are affected in Alzheimer’s disease (AD) and interact with amyloid-beta (Aβ) plaques. Apart from memory loss, depression is common in patients with AD. Electroconvulsive therapy (ECT) is an anti-depressive treatment that may stimulate microglia, induce neuroinflammation and alter the levels of soluble Aβ, but the effects of ECT on microglia and Aβ aggregation in AD are not known. We investigated the short- and long-term effects of ECT on neuroinflammation and Aβ accumulation. 5xFAD mice received either electroconvulsive stimulation (ECS n = 26) or sham treatment (n = 25) for 3 weeks. Microglia and Aβ were analyzed in samples collected 24 h, 5 weeks, or 9 weeks after the last treatment. Aβ plaques and microglia were quantified using immunohistochemistry. The concentration of soluble Aβ and cytokines was quantified using ELISA and levels of Aβ aggregates were measured with Western Blot. Microglial phagocytosis of Aβ in the hippocampus was evaluated by flow cytometry in Methoxy-X04 injected mice 24 h following the last ECS treatment. Y-maze and Elevated plus maze were performed to study behavior after 5 weeks. We could not detect any significant short- or long-term effects of ECS on Aβ pathology or neuroinflammation, but ECS reduced abnormal behavior in the Elevated Plus maze.

## Introduction

Alzheimer´s disease (AD) is the most common form of dementia, affecting around 30 million people worldwide (WHO 2016). AD is characterized by accumulation of amyloid-beta (Aβ) plaques and tau neurofibrillary tangles. Patients typically have a progressive neurodegeneration and hippocampus is one of the first regions to be affected, leading to cognitive dysfunctions. In addition to the abnormal high levels of Aβ in the brain of AD patients, the inflammatory status is also found to be altered in the AD brain^1^. Aβ can activate microglia and lead to production of cytokines, reactive species^2^ and pro-inflammatory factors with neurotoxic properties. Conversely, microglia might also be involved in neuroprotection due to its ability to clear Aβ fragments through phagocytosis^3^.

Even though cognitive dysfunction is a hallmark of AD, a majority of AD patients also suffer from other, non-cognitive symptoms such as depression and anxiety^4, 5^. These behavioral and psychological symptoms of dementia affect up to 80% of all AD patients^4^. Furthermore, depression has been shown to be a risk factor for being diagnosed with AD later in life^6–8^. Electroconvulsive therapy (ECT) is currently the most efficient way of treating patients with drug-resistant severe major depression^9, 10^. The treatment consists of administration of an electric current via temporal or frontotemporal electrodes, in order to induce a controlled seizure^11^. The therapeutic mechanisms of this treatment are not fully understood, even though it is known to induce proliferative changes in the brain, such as gliogenesis, angiogenesis and neurogenesis^11^. Further, ECT has been shown to affect microglial activity and phagocytosis^12, 13^. Regarding the effect on microglial cells, previous studies show inconsistent results, where some studies reveal that ECT may increase microglial activity^12, 14, 15^ while others show that it leads to a decrease^16^ or has no effect^17^.

A common side-effect of ECT is anterograde^18, 19^ or retrograde amnesia^20, 21^. Hippocampus is one of the major brain structures involved in the formation and storage of long-term memory^22^. For that reason, effects of ECT on hippocampal function have been thoroughly investigated^11, 23–28^. Nevertheless the effect of ECT on AD has not been fully investigated. Given the prevalence of depression in this group of patients, both prior to and after cognitive decline^6, 8^ it is of great interest to elucidate the effects of ECT on neuroinflammation and accumulation of Aβ.

The 5xFAD strain is a mouse model with a fast development of AD pathology, showing accumulation of Aβ plaques and signs of neuroinflammation as early as 2–3 month of age and cognitive dysfunctions already at 5 months of age^29–33^. This makes the 5xFAD a suitable mouse model to study the effects of ECT on the development of amyloid plaque load, microglial neuroinflammation and cognitive dysfunctions seen in AD patients. The aim of this study was to investigate the effects of ECT on neuroinflammation, cognition and Aβ pathology using 4–5.5 months-old 5xFAD mice.

## Results

### Increased neurogenesis confirms ECS treatment efficiency

To validate the efficiency of ECS treatment we measured the level of Doublecortin (DCX) in the dentate gyrus (DG), a well-established marker for immature neurons as a signature for neurogenesis, known to be highly upregulated following ECS treatment^34^. We detected a significant increase of DCX-positive cells in the DG in response to ECS compared to sham in the 24 h groups (Supplementary Fig. S1A,B, T-test *p* = 0.00016). After 5 weeks, ECS-treated mice demonstrated similar levels of DCX-positive cells as the 24 h sham group (Supplementary Fig. S1A,C, T-test *p* = 0.86), validating the known temporary effect of ECS on neurogenesis in our set up.

### No significant effects of ECS on Aβ plaques, aggregates and peptides

**Figure 1:**
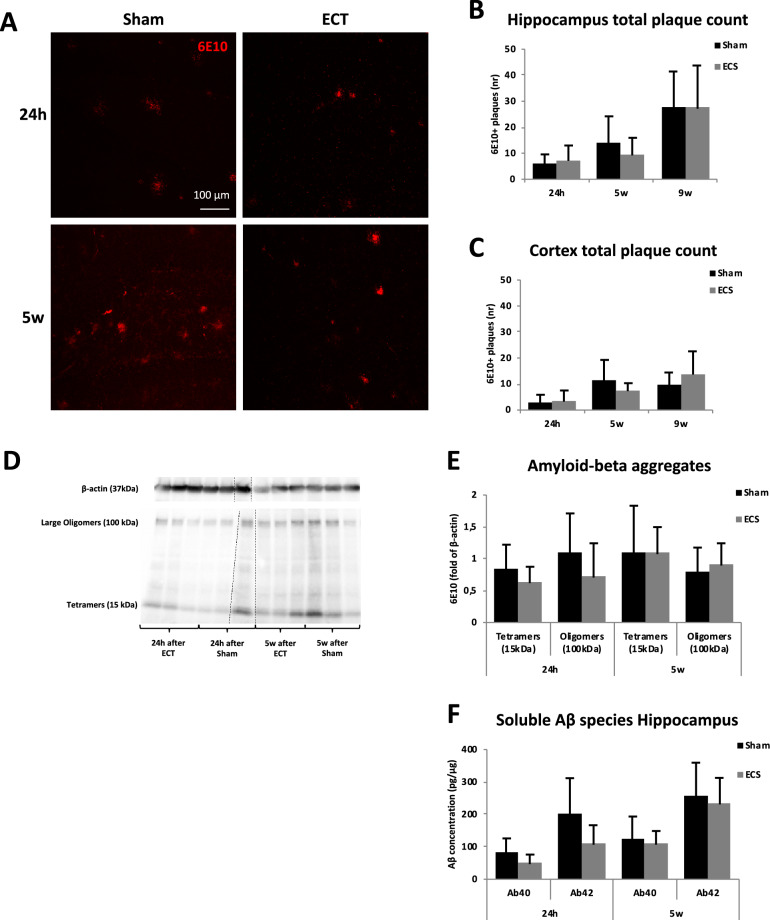
The effects of ECS on Aβ peptides, aggregates and plaques. (**A**) Representative confocal microscopy images (20 ×) of 6E10 staining in dentate gyrus. Scale bars represent 100 μm. Quantification of total plaque count from immunohistochemical staining in (**B**) hippocampus and (**C**) cortex. Images were analyzed with the free version ImageJ 1.52 (https://imagej.nih.gov/ij/). (**D**) Image of Western blot visualising tetramers and oligomers of Aβ aggregates. (**E**) Western Blot levels of Aβ aggregates in ECS and sham groups sacrificed 24 h and 5w after the last ECS session. The dotted lines on the gel indicate where western blot gel images have been cropped to align groups in a representative order. Original gels are available in Supplementary Figs. S3 and S4. (**F**) Concentration of soluble beta amyloid peptides (Aβ40 and 42) in ECS and sham groups sacrificed 24 h and 5 w after last ECS session. Bars represents mean values with error bars displaying SD. Analyses were done by unpaired two-tailed T-test (*p* ≤ 0.05*). For the 24 h groups, n = 4–8 for sham and n = 5–7 for ECS. For the 5 weeks groups, n = 8–10 for sham and n = 9 for ECS. For the 9 weeks groups, n = 5 for sham and n = 6 for ECS.

Immunohistochemical analysis of the amyloid β, using 6E10 staining, in hippocampus and cortex revealed no significant differences in the plaque number between ECS and sham at any time point (Fig. 1A,C,D, T-tests for hippocampus *p* = 0.51, *p* = 0.25 and *p* = 0.96 after 24 h, 5 w and 9 w respectively and for cortex *p* = 0.80, *p* = 0.18 and *p* = 0.34 after 24 h, 5 w and 9 w respectively). Likewise, thioflavin-S staining did not reveal any differences between ECS and sham (Supplementary Fig. S2, T-test for hippocampus *p* = 0.48 and for cortex *p* = 0.14). When stratifying on sex, the female ECS group had a significantly lower 6E10 plaque number in the cortex compared to the sham group after 5 weeks (*p* = 0.02, Supplementary Table S2). Apart from that, no other differences between ECS and sham were found for any of the stainings when analyzing females and males separately (Supplementary Table S1, S2, S4, and S5).

Using Western Blot, we analyzed and quantified the concentration of soluble and insoluble Aβ aggregates, including oligomers and protofibrils in hippocampus after 24 h and 5w. We detected two relatively strong bands, one at 15 kDa representing tetramers and one at 100 kDa representing large oligomers or small protofibrils. No significant differences could be detected between the sham and ECS groups (Fig. 1B,E and Supplementary Figs. S3 and S4, T-tests for tetramers *p* = 0.27 and *p* = 0.98 after 24 h and 5 w respectively and for oligomers *p* = 0.28 and *p* = 0.48 after 24 h and 5 w respectively). ECS did not have any significant effects on the concentration of soluble Aβ40 and Aβ42 peptides in hippocampus after 24 h and 5 w as measured with ELISA (Fig. 1F, T-tests for Aβ40 *p* = 0.18 and *p* = 0.65 after 24 h and 5 w respectively and for Aβ42 *p* = 0.14 and *p* = 0.63 after 24 h and 5 w respectively). No differences between ECS and sham were found when analyzing females and males separately (Supplementary Table S1).

### No significant effect of ECS on the amount of microglia and cytokine levels

**Figure 2:**
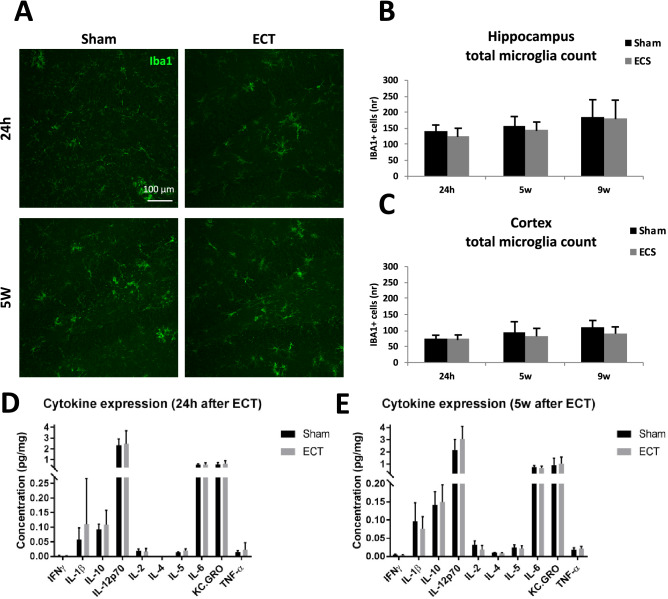
Effects of ECS on microglial activity. (**A**) Representative confocal microscopy images (20 × visualising Iba1 staining in dentate gyrus. Scale bars represent 100 μm. Quantification of microglial cells in ECS and sham groups sacrificed 24 h, 5 w and 9 w after last ECS session in (**B**) hippocampus and (**C**) cortex. Images were analyzed with the free version ImageJ 1.52 (https://imagej.nih.gov/ij/). Levels of pro/anti-inflammatory cytokines in the hippocampus (**D**) 24 h and (**E**) 5 w after last ECS session. Bars represents mean values with error bars displaying SD. Analyses were done by unpaired T-test (*p* ≤ 0.05*). For the 24 h groups, n = 8 for sham and n = 7 for ECS. For the 5 weeks groups, n = 9 for sham and n = 9 for ECS. For the 9 weeks groups, n = 5 for sham and n = 6 for ECS.

Immunohistochemical analysis of the number of microglia in hippocampus and cortex revealed no significant differences between ECS and sham for any of the time points investigated (Fig. 2A–C, T-tests for hippocampus *p* = 0.16, *p* = 0.29 and *p* = 0.95 after 24 h, 5 w and 9 w respectively and for cortex *p* = 0.96, *p* = 0.42 and *p* = 0.23 after 24 h, 5 w and 9 w respectively) Further, no differences between ECS and sham were found when analyzing females and males separately (Supplementary Tables S1, S2, S4, and S5). Moreover, we could not detect any significant differences in cytokine concentrations in hippocampus between ECS and sham after 24 h or 5 w (Fig. 2D,E, T-tests after 24 h *p* = 0.83, *p* = 0.42, *p* = 0.46, *p* = 0.82, *p* = 0.68, *p* = 0.07, *p* = 0.90, *p* = 0.48 and *p* = 0.42 for INFγ, IL-1β, IL-10, IL-12p70, IL-2, IL-5, IL-6, CXCL and TNF-α respectively and after 5 w *p* = 0.20, *p* = 0.61, *p* = 0.68, *p* = 0.06, *p* = 0.06, *p* = 0.47, *p* = 0.44, *p* = 0.17, *p* = 0.62 and *p* = 0.33 for INFγ, IL-1β, IL-10, IL-12p70, IL-2, IL-4, IL-5, IL-6, CXCL and TNF-α respectively).

### ECS treatment does not affect microglial phagocytosis of amyloid beta

**Figure 3:**
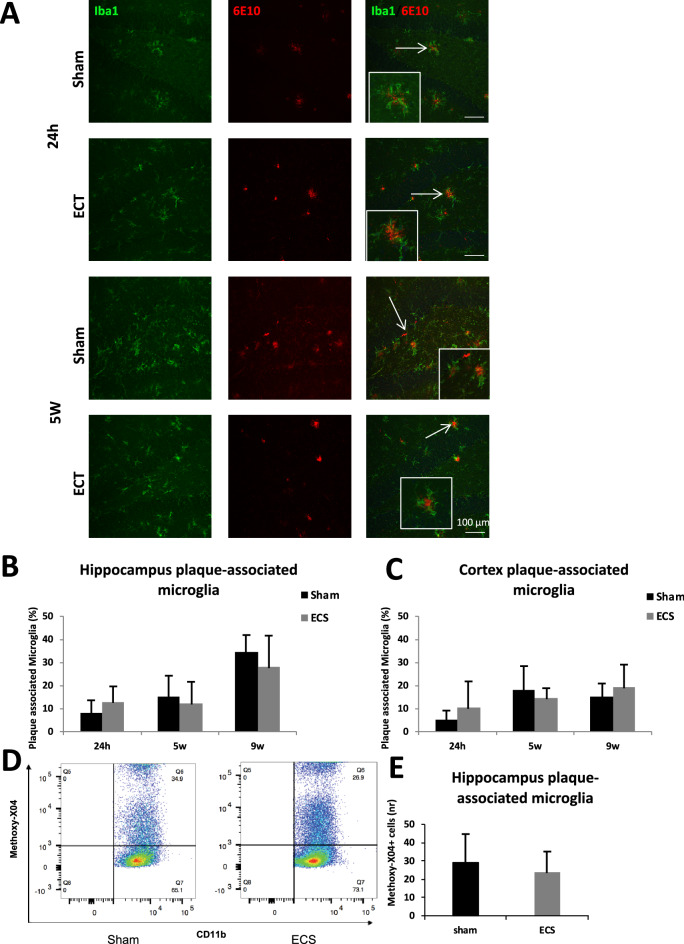
Effects of ECS on microglial interaction with amyloid plaques. (**A**) Representative confocal microscopy images (20 ×) visualising DAPI, Iba1 and 6E10 stainings in dentate gyrus. Scale bars represents 100 μm. Percentage of plaque associated microglia after 24 h, 5 w and 9 w in (**B**) hippocampus and (**C**) cortex. (**D**) Representative scatter plots of CD11b and Methoxy-X04 flow cytometry after 24 h and (**E**) quantification of the percentage of methoxy-X04 positive microglial cells. Bars represents mean values with error bars displaying SD. Flow cytometry was analysed with the licenced version of FlowJo 10.4.2 (https://www.flowjo.com/solutions/flowjo/downloads). Analyses were done by unpaired T-test (*p* ≤ 0.05*). For the 24 h groups, n = 7 for sham and n = 8 for ECS. For the 5 weeks groups, n = 9 for sham and n = 9 for ECS. For the 9 weeks groups, n = 5 for sham and n = 6 for ECS.

Immunohistochemical analysis of the ratio of plaque-associated microglial cells in hippocampus and cortex revealed no differences between ECS and sham after 24 h and 5 w (Fig. 3A–C, T-tests for hippocampus *p* = 0.15, *p* = 0.52 and *p* = 0.49 after 24 h, 5 w and 9 w respectively and for cortex *p* = 0.24, *p* = 0.33 and *p* = 0.43 after 24 h, 5 w and 9 w respectively.). No differences between ECS and sham were found when analyzing females and males separately (Supplementary Table S1 and S2). Furthermore, we could not detect any differences in methoxy-X04 positive (Aβ containing) microglia measured by flow cytometry between ECT and sham 24 h after the last ECS session (Fig. 3D,E, T-test, *p* = 0.60).

### ECS treatment affects exploratory behavior but not working memory

**Figure 4:**
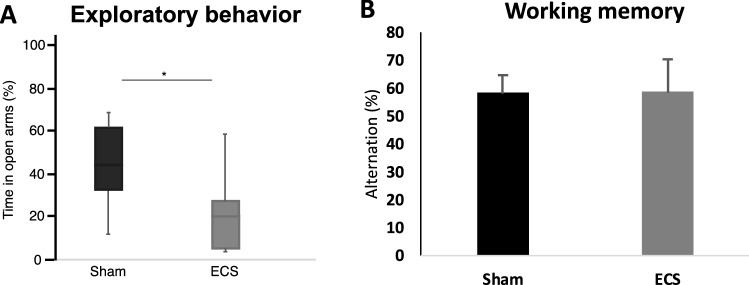
The effect of ECS on behavioural outcome. (**A**) Exploratory behaviour measured in Elevated Plus Maze. Box plots represent the median values for each group with interquartile ranges and error bars indicating the minimum and maximum. (**B**) Working memory measured as spontaneous alternation in Y-maze after 5 weeks. Bars represent mean values with error bars displaying SD * represents *p* ≤ 0.05 in Mann–Whitney U-test. For sham n = 7 and 6, for ECS n = 9 and 10 for Elevated plus maze and Y-maze respectively.

Using open field test, we found no differences in locomotor behavior between ECS and sham groups at baseline (prior to ECS treatment, data not shown). In elevated plus maze, mice treated with ECS showed a reduction in exploratory behavior compared to sham mice 5 weeks after last ECS session (Fig. 4A, Mann–Whitney U-test, *p* = 0.04). Further, no differences in working memory as measured with Y-maze spontaneous alternation test were seen between ECS and sham after 5 weeks (Fig. 4B, T-test, *p* = 0.86). In addition, no differences between ECS and sham were found when analyzing females and males separately (Supplementary Table S3). For hindlimb clasping, no significant differences could be detected clasping between ECS and sham-treated mice for any of the time-points analyzed (Supplementary Fig. S5. Mann–Whitney U-tests, *p* = 0.30 for week 0, *p* = 0.40 for week 5 and *p* = 0.34 for week 8).

## Discussion

Our study investigated the short- and long-term effects of ECS treatment on development of behavioral deficits, amyloid pathology and neuroinflammation in the 5xFAD mouse model of AD. We found no effects of ECS on Aβ pathology, microglial activity or cognitive function. ECS treatment did however reduce exploratory behavior, potentially suggesting a normalization of aberrant behavior in the 5xFAD mouse model of AD.

Although we could not detect any significant effects of ECS on different Aβ-species with ELISA in this study, ECT has been shown to affect the levels of Aβ-42 in cerebrospinal fluid (CSF) of depressive patients^35^. Aβ42 is considered to be the most pathogenic with the highest tendency to aggregate and form amyloid plaques^36^. Over time, the brain of a patient with AD accumulates more Aβ42, whereas the level of Aβ42 in the CSF decreases^37^. Thus Aβ-levels in CSF are inversely correlated with those found in the brain^38, 39^. Interestingly this Aβ peptide alteration pattern is very similar to what is observed in patients suffering from depression, suggesting a possible link between depression and increased risk for future development of AD^35^. In fact, Kranaster et al. showed that increased levels of Aβ42 in CSF was only found in patients who had an antidepressant response to ECT^35^. In our Aβ ELISA analysis of the hippocampus, we observed a tendency (*p* = 0.13) for the average concentrations of Aβ-42 to be lower after ECS treatment, especially in the short-term group. Still, the differences between our groups were far from being statistically significant, possibly due to the very large variations within each group. If these differences in average Aβ-42 concentrations would have been significant they would had been in line with the results in the above mentioned study^35^ due to the inverse relationship between Aβ-42 concentrations in the brain and CSF.

Unlike previous research^12^, no significant effects of ECS treatment on microglial cell number or levels of cytokines could be detected in our study. Indeed, studies investigating the effects of ECS in rats indicated an increase in the number of microglia as well as increased levels of pro-inflammatory cytokines IL-1β and TNF-α^40, 41^. Further, other studies also show that ECS might lead to increased expression of phagocytic markers on microglia^12^. Thus, we aimed to investigate the effect of ECS on microglial interaction with the Aβ plaques and also phagocytosis of Aβ specifically. We could not detect any significant differences in the percentage of plaque associated microglia between groups. However, this might be due to the very large variations between animals within the same group. Interestingly, the average percentage of plaque associated microglia seemed to increase 24 h following ECS compared to sham, but to be lower than sham 5 weeks after ECS both in hippocampus and cortex. Still, due to large variations, these differences are far from being significant and potential effects of ECS could not be concluded. Moreover, no significant differences in microglial phagocytosis of Aβ were found between ECS and sham as measured by Methoxy-X04 positive microglia using flow cytometry 24 h after last ECS session.

We could not detect any effects of ECS on working memory as measured with Y-maze spontaneous alternation test. This does not exclude the possibility of ECS to transiently impair working memory in this model, since the test was conducted 5 weeks following last treatment session. Also, our mice were 6–7 month old when conducting this test and it is well-known that the 5xFAD model displays working memory deficits already at 4–5 months of age^30, 42^. Hence, we could not detect any indications for ECS treatment to aggravate such deficits in a long-term perspective. We did however detect a significant reduction in exploratory behavior in the Elevated Plus Maze test compared to sham 5 weeks after the last ECS session. It has been shown in several studies that the 5xFAD model develops increased exploratory behavior in the Elevated Plus Maze test^43^ and that this behavior correlates with the accumulation of Aβ in the brain^43–45^. This increase in exploratory behavior has been suggested to reflect the dis-inhibitory tendencies in AD patients^44^. Hence, reduced exploratory behavior of 5xFAD mice in this test might be interpreted as an attenuation of this disinhibition in this mouse model as we previously reported^46^.

Limitations of the study include that the 5xFAD model we used in this study has a very rapid progression of the AD pathology. It is also possible that the large variations within each group is somewhat reflecting the variations that can occur between 5xFAD animals due to its rapid progression. Further, we only studied the effect of ECS at three discrete timepoints, thus we can never exclude the risk of missing significant effects occurring at other timepoints. However, we selected this mouse model since it is a robust AD model with a clear neuroinflammatory component. We included both short-term (24 h) and long-term(5w and 9w) time points as the effects of ECS on microglia have been shown to be rather short-lasting and to occur within the first hours to days following treatment^12^. Additionally, we confirmed that the ECS treatment was properly applied as it resulted in tonic–clonic seizures and an induction of neurogenesis 24 h following ECS treatment (a well-known effect of ECS when applied efficiently). Moreover, we focus our extensive investigations on the hippocampus since this is the region mostly investigated in this model and also the region best characterized with regards to the effect of electroconvulsive stimulation in wildtype settings. Hence, it is possible that the electroconvulsive stimulation affected cytokine levels and different amyloid species in other regions of interest, such as the cortex. Nevertheless, our results from the 6E10, thioflavin-S, and Iba1 staining in the cortex did not show any effect of the treatment, making it less probable that the electroconvulsive stimulation affects amyloid accumulation or microglial neuroinflammation in that region.

Taken together, we could not detect any significant effects of ECS treatment on cognition, neuroinflammation, Aβ pathology nor microglial phagocytosis of Aβ in our 5xFAD mouse model of AD. This lack of detrimental effects is a promising indication for patients in need of this antidepressant treatment. Due to large variations within our experimental groups we cannot exclude any effects and more research will be needed to fully investigate the effects of ECT on AD pathology in this group of patients.

## Material and methods

### Mouse model

All experiments were approved by the local ethical committee at Lund University (D .nr 5.8.18–01 107/2018) and performed in accordance with the Directive of the European Parliament as well as in compliance with the ARRIVE guidelines. We used 51 in-house bred 5xFAD mice (originally from Jackson Laboratory) aged 15–22 weeks and weighing 20-33 g at the start of the experiment. The mice were housed 2–5 animals per standard laboratory cage, with sawdust bedding and free access to water and food. The holding room had a 12:12 h light–dark cycle.

### Experimental outline

**Figure 5:**
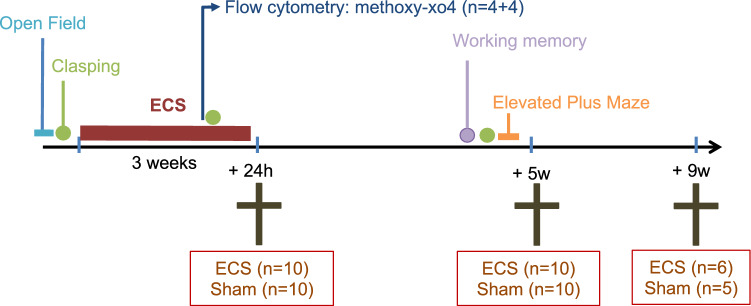
Experimental outline. Timeline of the study presenting the individual steps before and after ECS until animals were sacrificed.

An overview of the experimental outline can be seen in Fig. 5. Briefly, mice (n = 51 were divided in two major groups; one that was subjected to electroconvulsive stimulation (ECS, n = 26) and the other to sham treatment (n = 25). To study both the short- and long-term effects of ECS, the treatment groups were further divided into three different subgroups with follow-ups 24 h (short term, n = 10 + 10), 5 weeks (long term, n = 10 + 10) and 9 weeks (long term, n = 6 + 5) after the last treatment. The 24 h group was used for flow cytometry and the 5w group was used for behavioral testing.

### Electroconvulsive Stimulation treatment

Treatment with ECS was initiated in 15–22 weeks old mice. All the mice were lightly anesthetized (1.5% isofluorane) and the animals receiving ECS treatment were given electrical shocks (45 mA for 1 s, 100 Hz, 0.9 ms/pulse, using 57,800–01 ECT Unit, Ugo Basile, Gemonio, Italy) via electrodes attached to the ears, resulting in tonic–clonic seizures lasting approximately 10 s (indicating time from seizure on-set to muscle relaxation). Control mice were sham-treated, i.e. handled identically to the ECS mice except that no current was applied. The mice received ECS or sham treatment 3 times a week, with a total of 9 sessions.

### Collection of samples

The acute groups were sacrificed 24 h after the last ECS session and the long-term groups were sacrificed 5 and 9 weeks after the last ECS session. Mice were first anesthetized via inhalation of isoflurane (5% isoflurane in oxygen), thereafter euthanized by transcardial perfusion with ice-cold phosphate-buffered saline (PBS). The brains were dissected and the right hemisphere was fixated in 4% paraformaldehyde in PBS for 24 h and washed with PBS before they were stored in 30% sucrose solution at 4 °C. Using a microtome, 30µm coronal brain sections were cut and stored in a cryoprotectant solution (30% sucrose + 30% ethylene glycol in PBS) at − 20 °C^47^. From the left hemisphere, hippocampus was dissected and snapfrozen in crushed dry ice and stored at − 80 °C until homogenization for analysis with Western Blot and ELISA.

### Methoxy-X04 injections and microglial cell isolation

In order to analyze the microglial phagocytosis of Aβ, a subset of mice (n = 4 + 4) within the 24 h groups were injected intraperitoneally with Methoxy-X04 2 h prior to sample collection. Hippocampus from these mice were kept in ice cold PBS and immediately used for isolation of microglial cells. The microglial cell isolation procedure was performed according to an established protocol from MACS Miltenyi Biotec; Neural tissue dissociation kit and Cell separation by CD11b (microglia) microbeads.

### Flow cytometry analysis

Fc receptors on isolated microglia were blocked with anti-CD16/CD32 antibody (BD Bioscience, 1:100). Thereafter, samples were incubated with anti-CD11b-APC (Biolegend, 1:800) and anti-CD45-PE (BD Bioscience; 1:400). Compensation was made with single stainings and gating was determined by proper negative isotype stained controls. Dead cells were excluded by a viability staining, PI (propidium iodide) (BD Bioscience). Flow cytometry was performed in a FACSArial III cytometer (BD Biosciences) and FACS Diva software (BD Biosciences). Microglia were identified by CD11b + and CD45 + expression and ten thousand events were recorded. Phagocytic microglia were recognized with Methoxy-X04 (Supplementary Figs. S6 and S7). FlowJo 10.4.2 software (Flowjo, LLC) was used for data analysis.

### Immunohistochemistry

#### Quantification of plaques and microglia

Immunohistochemistry was performed as described previously^48^, with minor modifications. Briefly, 30 µm coronal sections were mounted on glass slides (Menzel Gläser, Superfrost Plus, Thermo Scientific) and stained with 6E10 (1:500, mouse, Aβ 1–16, BioLegend) and Iba1 (1:300, rabbit, Wako). Secondary antibodies were anti-mouse (1:1000, wavelength 555, Alexa Fluor, Invitrogen) and anti-rabbit (1:1000, wavelength 488, Alexa Fluor, Life Technologies). The cortex (parietal association region together with the somatosensory region) and hippocampus (dentate gyrus (DG) and cornu ammonis 2 and 3 (CA2 and CA3) were analyzed in three sections per brain using an epifluorescence microscope (Olympus BX43, LRI, SE, Nikon Eclipse 80i). The number of microglia, amyloid plaques and plaque-associated microglia were analyzed using FIJI (ImageJ) software. Plaque associated microglia were defined as microglia (Iba1+) either co-localizing with the plaque (6E10+) or localized around/next to the plaque. Thioflavin-S staining and analysis were performed as described previously^46^ and in Supplementary Methods.

#### Quantification of neurogenesis

Neurogenesis measurements were performed as previously described^48^ in Supplementary Methods.

### Brain tissue homogenization and protein concentration determination

Frozen hippocampal samples were thawed on ice. The samples were homogenized and separate fractions, S1 (extracellular material), S2 (intracellular debris), and S3 (smaller aggregates) were generated and collected according to previously described protocol^48^. Protein concentrations in the collected homogenized fractions were measured using BCA kit ^48^. All fractions collected were stored at − 20 °C until analysis.

### Amyloid beta aggregates in Western Blot

Western blots were used to analyze the amount of amyloid oligomers and tetramers in the hippocampus S3 fraction, containing smaller amyloid aggregates. The western blots were performed as previously described^48^ with the addition of a 5 min membrane incubation in boiling PBS after the transference. Membranes were stained for 6E10 (1:5000, mouse, Aβ 1–16, BioLegend) and secondary anti-mouse (1:10,000, Peroxidase Vector laboratories). Membranes were developed using the ECL clarity developing kit (BioRad) following the manufacturer’s instructions.

### Multiplex cytokine ELISA

The MSD Mouse Proinflammatory V-Plex Plus kit (INF-γ, IL-1β, IL-12p70, IL-2, IL-4, IL-5, IL-6 IL-10, CXCL1, TNF-α; K15012C, Mesoscale) was used to measure the concentrations of different cytokines in the pooled S1 and S2 fractions of homogenized hippocampus (25 µl/sample) by using a QuickPlex SQ120 (Mesoscale Discovery, Rockville, USA) Plate Reader according to the manufacturer’s instructions and as described previously^46^. The MSD Discovery Workbench software was used to analyze the data. The concentrations were normalized to protein concentrations measured with the Bradford assay.

### Aβ ELISA

The concentration of different Aβ species in the pooled S1 and S2 fractions of homogenized hippocampus were measured in the MSD (Aβ38, Aβ40 and Aβ42; K15199G-1, Mesoscale) using QuickPlex SQ120 (Mesoscale Discovery, Rockville, USA) Plate Reader according to the manufacturer’s instructions. The recorded data was analyzed using MSD Discovery Workbench software. Samples were diluted to acquire a total of 4.5 µg protein/sample.

### Behavioral tests

#### Open field test

An open field test was used to control for baseline locomotion prior to the ECS treatment to assure that mice in different groups did not differ in baseline locomotor behavior as described in Supplementary Methods.

#### Clasping scoring

Hindlimb clasping tests were performed as described in Supplementary Methods.

#### Y-maze spontaneous alternation test

Y-maze spontaneous alternation test was performed 5 weeks after the last ECS session to examine working memory, using a Y-shaped arena (35 cm × 5 cm/arm) as described before^49^. Bedding material from the home cage of the mouse in the test was placed in the arms of the maze for familiarity and thus increase the likelihood for it to explore the maze. To prevent odor bias, bedding material was removed and the maze was cleaned with ethanol followed by water before the next mouse was introduced to the maze. The mouse was placed in the first arm facing the walls and then left alone to freely explore the three arms for 8 min. Percentage of spontaneous alternation was defined as consecutive entries in 3 different arms (A, B, and C), divided by the number of possible alternations (total arm entries minus 2). A lower degree of spontaneous alternation indicates working memory dysfunction. Mice with less than 5 arm entries during the 8-min trial were excluded from this analysis.

#### Elevated plus maze test

To examine exploratory behavior, mice in the 5w group were subjected to the elevated plus maze test (Fig. 1). The elevated plus maze apparatus consisted of two open arms and two closed arms (29 cm × 6 cm). The entire maze was elevated 40 cm from the floor. Each mouse was placed in the center of the maze with the head facing towards the open arm. During a 5 min test, the behavior was recorded and the time spent in the open arms was manually evaluated afterwards. Spending time in the open arms is interpreted as exploratory behavior.

### Statistical analysis

Statistical analyses were conducted in excel and SPSS (v.25). To compare the differences between ECS and sham on cognition, the level of plaque deposition, microglial presence and phagocytosis, unpaired two-tailed T-tests were used. Data from clasping tests and the elevated plus maze test were not normally distributed. Therefore non-parametric tests were used for this data. Exploratory behavior in the elevated plus maze was analyzed with the Mann–Whitney U-test. Friedman tests were used to analyze the evolution of clasping behavior over time within groups. For specific time-points of the clasping tests, groups were compared with Mann–Whitney U-tests. Additionally, for amyloid plaques and proteins, microglial quantity, and behavioral tests, data were also stratified by sex to investigate potential differences between females and males when the number of datapoints within each group allowed. P-values below 0.05 were considered statistically significant.

## Supplementary Information

Supplementary Information.
