# Supplementary material for: The effect of electroconvulsive therapy on neuroinflammation, behavior and amyloid plaques in the 5xFAD mouse model of Alzheimer’s disease
Source: Sci Rep. 2021 Mar 1;11:4910. doi: 10.1038/s41598-021-83998-0 (PMC7921388; doi:10.1038/s41598-021-83998-0)
Supplement: Supplementary file 1 — Supplementary Information. [file 41598_2021_83998_MOESM1_ESM.pdf]

Supplementary Information

The effect of electroconvulsive therapy on neuroinflammation, behavior and amyloid plaques in the 5xFAD mouse model of Alzheimer's disease

Martina Svensson, Gustaf Olsson, Yiyi Yang, , Sara Bachiller, Maria Ekemohn, Joakim Ekstrand, Tomas Deierborg

Supplementary figures

Figure S1

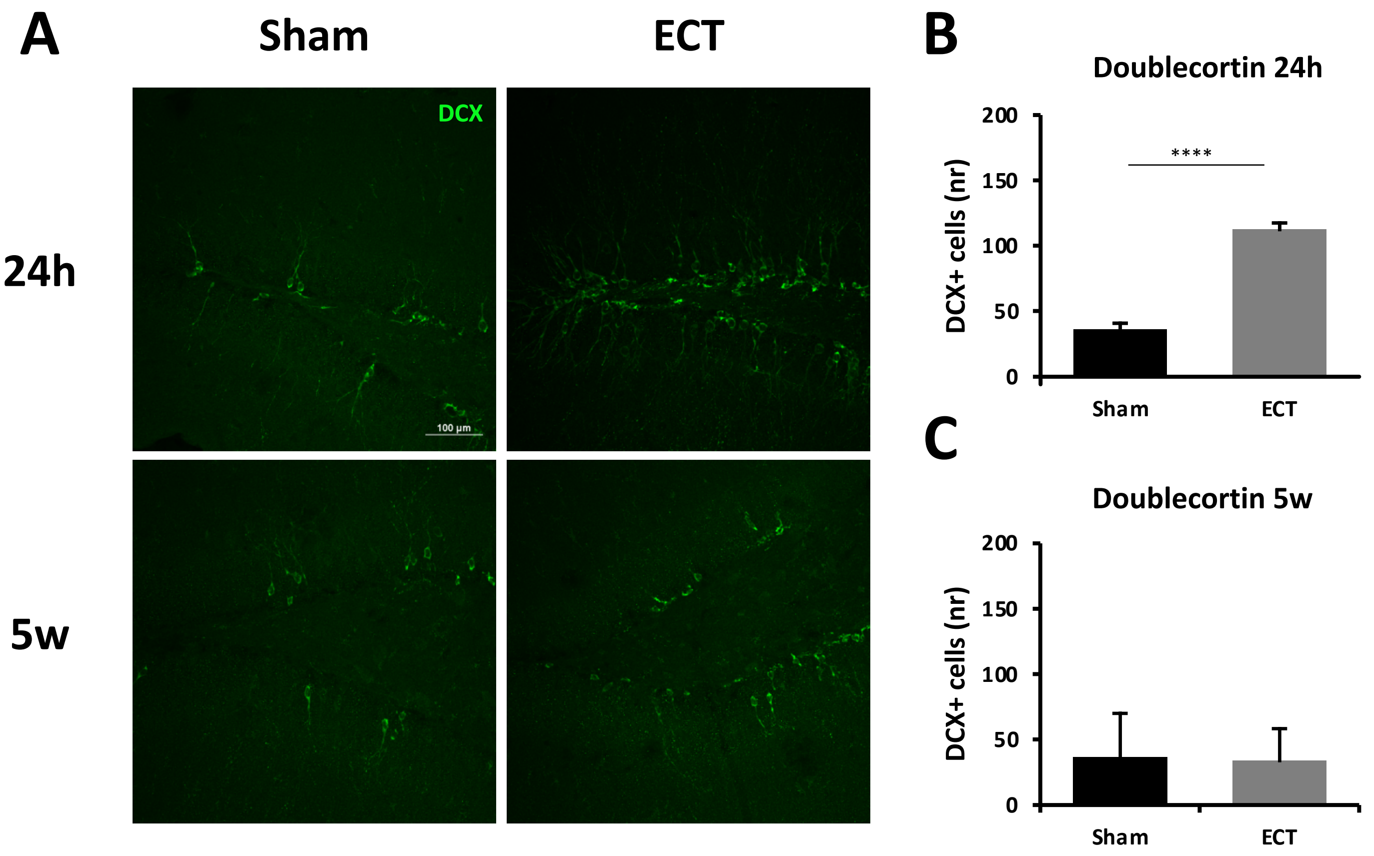

Fig S1. Increase of Doublecortin (DCX) positive cells in dentate gyrus (DG) as a measure of neurogenesis.
(A) Representative confocal microscopy images (20x) visualising doublecortin (DCX) in dentate gyrus. Scale bars represent 100 μm (B) Quantification of DCX+ cells in ECS and sham groups 24h and (C) 5w after last ECS session. Bars represents mean values with error bars displaying standard deviation (SD). Analyses were done by unpaired two-tailed T-test with statistical significance ($p \leq 0.05$*, ≤0.01**, ≤0.001***, ≤0.0001****).

Figure S2

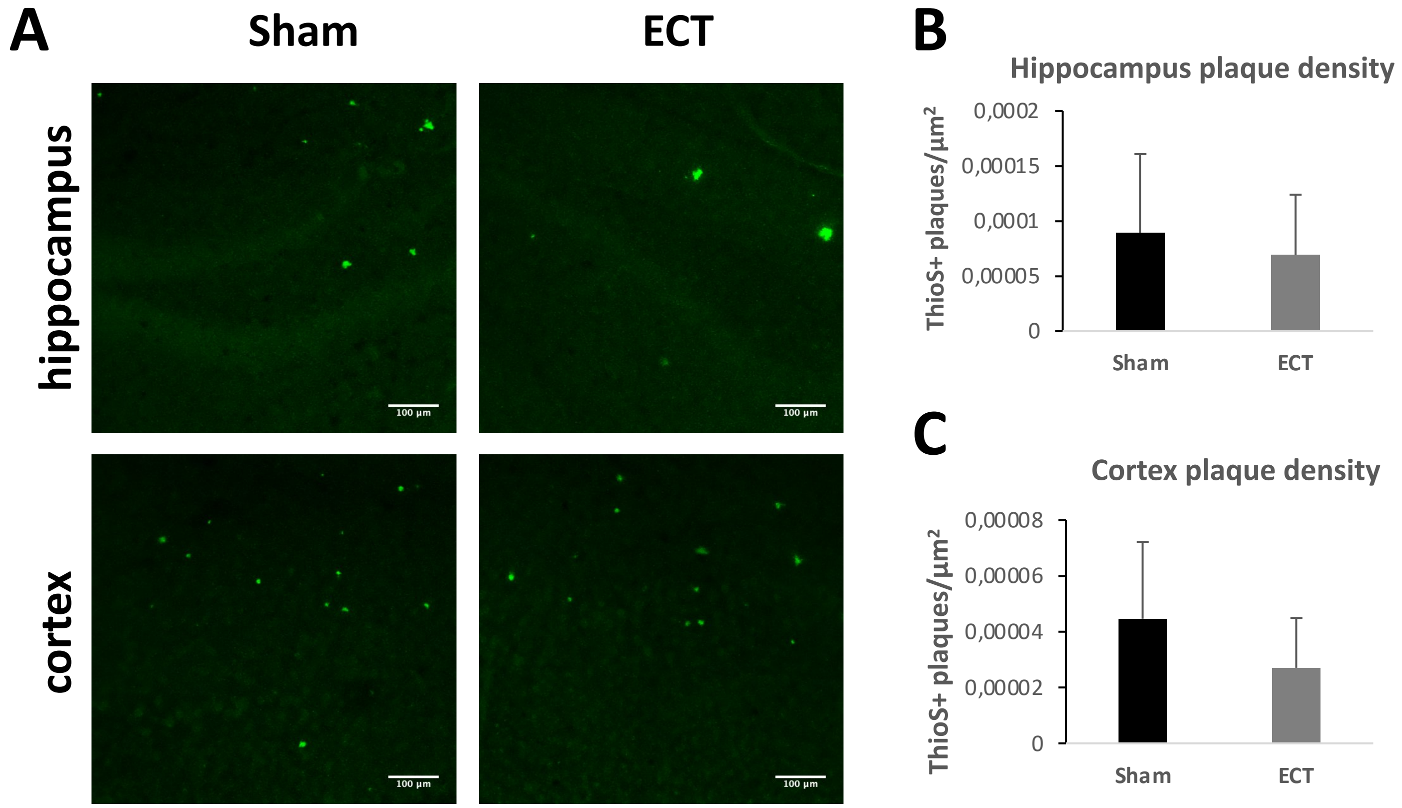

Fig S2. Effects of ECS on fibrillary amyloid plaques

(A) Representative microscopy images (10x) visualising thioflavin-S in hippocampus and cortex. Scale bars represent 100 µm. Quantification of plaque density in hippocampus (B) and cortex (C) 5w after last ECS session. Bars represents mean values with error bars displaying standard deviation (SD). T-test with statistical significance $p \leq 0.05$ revealed no significant difference for any of the regions analyzed.

Figure S3

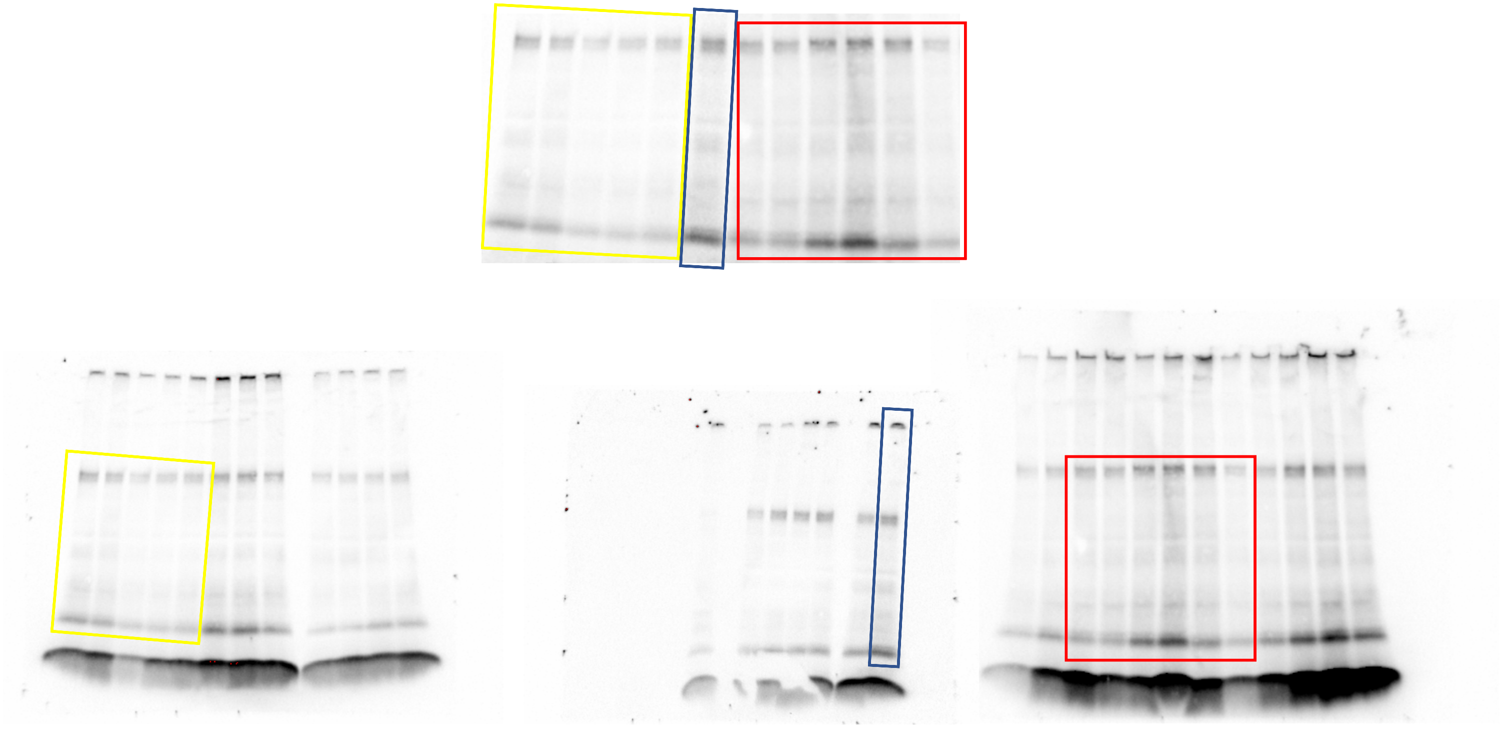

Fig S3. Aβ aggregates in original Western blot gels.

From left to right

Yellow: 24h ECT (3 samples)

24h Sham (2 samples)

Blue: 24h Sham (1 sample)

Red: 5w ECT (3 samples)

5w Sham (3 samples)

Figure S4

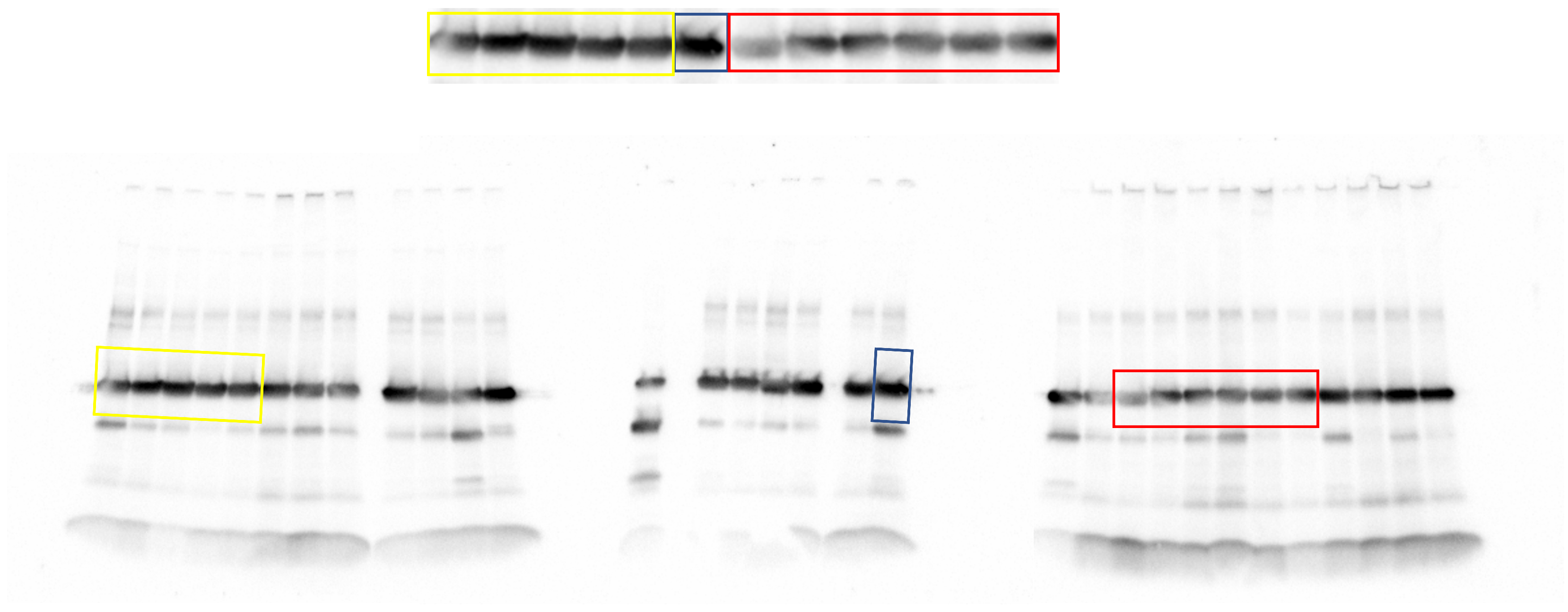

Fig S4. β-actin in original Western blot gels.

From left to right

Yellow: 24h ECT (3 samples)

24h Sham (2 samples)

Blue: 24h Sham (1 sample)

Red: 5w ECT (3 samples)

5w Sham (3 samples)

Figure S5

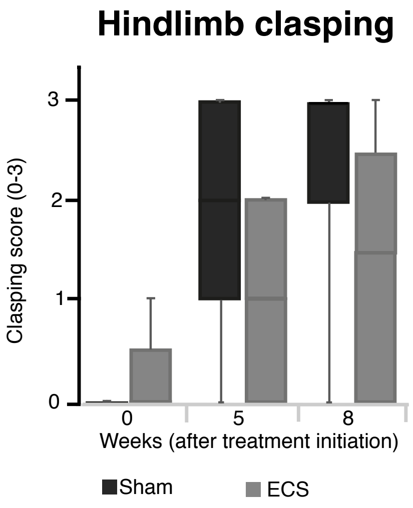

Fig S5. The effect of ECS on hindlimb clasping

Development of hindlimb clasping over time. Box plots represent the median values for each group with interquartile ranges and error bars indicating the minimum and maximum. There were significant changes in hindlimb clasping scores over time within both treatment groups (week 0, 5 and 8, Friedmans tests, p=0.02 for sham and p=0.002 for ECS), but there were no significant differences in clasping score between groups for any of the time-points analyzed (Mann-Whitney U-tests, p=0.30 for week 0, p=0.40 for week 5 and p=0.34 for week 8).

Figure S6

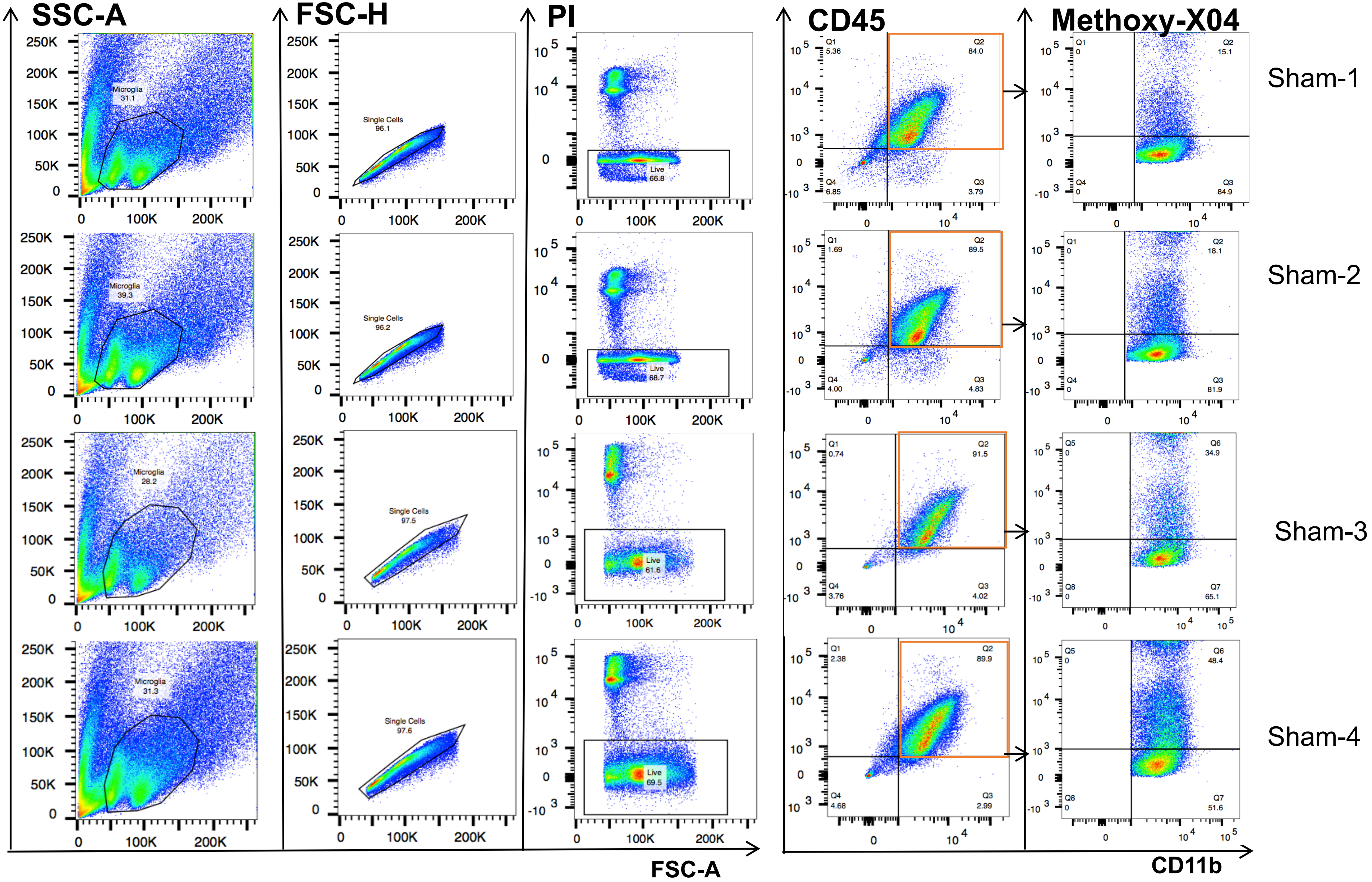

Fig S6. Gating strategy for the flow cytometry experiments

Live cells were selected with propidium iodide- (PI) negativity, and microglia with CD45 and CD11b. Finally, phagocytic microglia was counted as the number of methoxy-X04-positive cells.

Figure S7

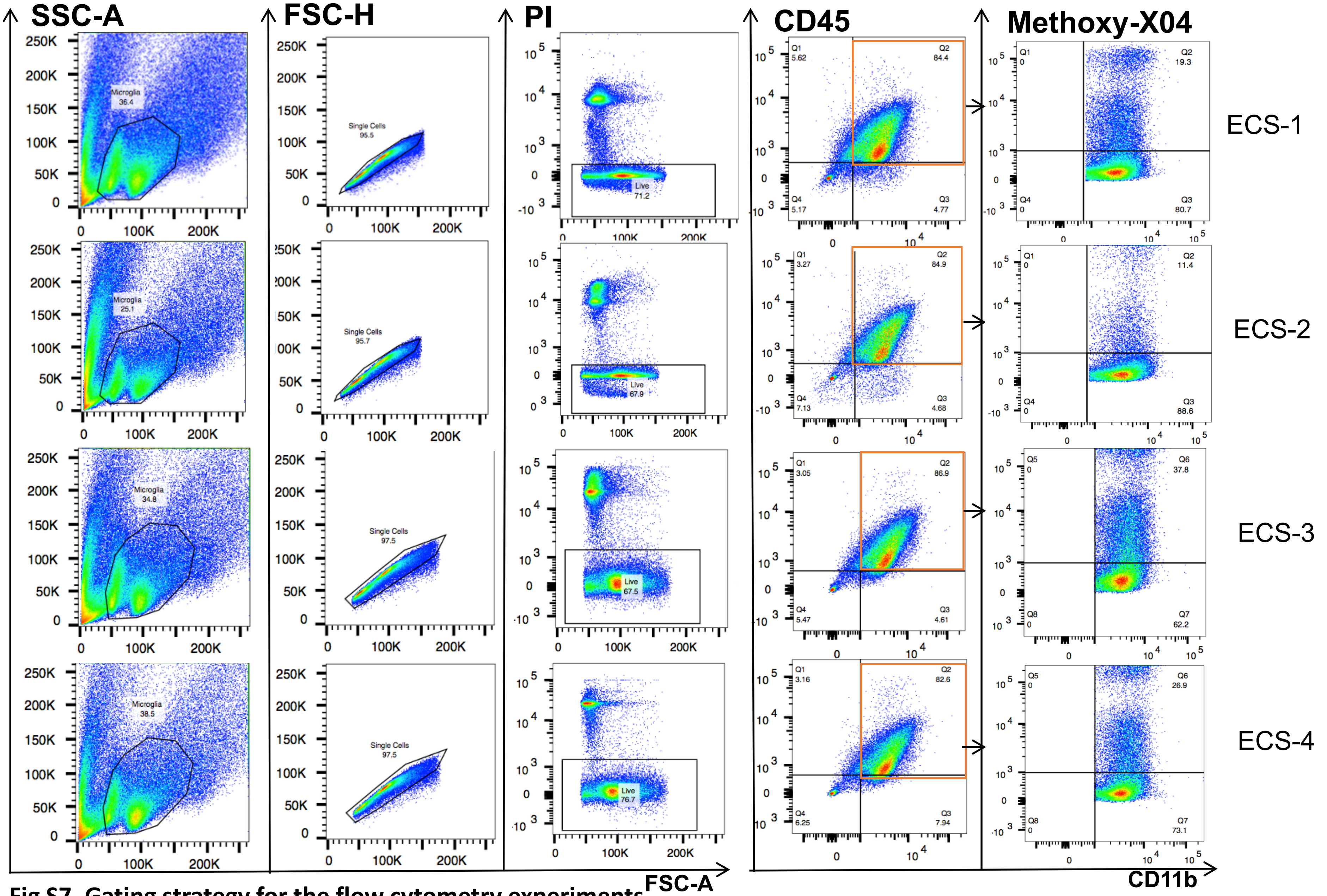

Fig S7. Gating strategy for the flow cytometry experiments

Live cells were selected with propidium iodide- (PI) negativity, and microglia with CD45 and CD11b. Finally, phagocytic microglia was counted as the number of methoxy-X04-positive cells.

Supplementary Tables

Table S1. Hippocampus 5 weeks after treatment

	Females			Males		
	Sham Mean (S.D)	ECS Mean (S.D)	p-value, T-test	Sham Mean (S.D)	ECS Mean (S.D)	p-value, T-test
6E10 (plaque nr)	22.8 (6.2)	16.8 (2.0)	0.18	8.8 (6.2)	8.1 (2.5)	0.86
ThioS (plaques/μm^2)	0.00015 (0.000056)	0.00013 (0.000037)	0.68	0.000064 (0.000052)	0.000046 (0.000018)	0.54
Amyloid Tetrameres (fold of β-actin)	1.77 (0.51)	1.49 (0.27)	0.43	0.83 (0.44)	1.06 (0.19)	0.38
Amyloid Oligomeres (fold of β-actin)	1.18 (0.16)	1.22 (0.17)	0.48	0.68 (0.36)	0.87 (0.29)	0.45
Aβ40 (pg/μg)	171.9 (85.0)	130.2 (34.7)	0.48	98.5 (53.1)	121.0 (25.3)	0.47
Aβ42 (pg/μg)	331.8 (120.6)	237.1 (40.9)	0.27	209.4 (78.7)	285.0 (62.7)	0.18
Iba1+ microglia (nr)	173.1 (19.0)	132.2 (43.3)	0.15	148.9 (35.4)	147.1 (14.5)	0.93
Plaque-associated microglia (%)	23.3 (3.6)	23.3 (5.5)	0.99	9.8 (5.8)	8.7 (3.4)	0.76

Supplementary Table S1. S.D= standard deviation

Table S2. Cortex 5 weeks after treatment

	Females			Males		
	Sham Mean (S.D)	ECS Mean (S.D)	p-value, T-test	Sham Mean (S.D)	ECS Mean (S.D)	p-value, T-test
6E10 (plaque nr)	15.3 (8.4)	10.7 (2.1)	0.02	9.4 (7.2)	5.9 (1.5)	0.38
ThioS (plaques/µm^2)	0.000064 (0.000017)	0.000049 (0.000046)	0.18	0.000038 (0.000031)	0.000019 (0.000009)	0.31
Iba1+ microglia (nr)	114.3 (25.1)	103.8 (26.2)	0.62	87.3 (30.6)	76.8 (13.0)	0.55
Plaque-associated microglia (%)	20.4 (9.7)	16.8 (6.8)	0.61	16.2 (13.3)	13.9 (3.2)	0.75

Supplementary Table S2. S.D= standard deviation

Table S3. Behavior 5 weeks after treatment

	Females			Males		
	Sham Mean (S.D)	ECS Mean (S.D)	p-value, T-test	Sham Mean (S.D)	ECS Mean (S.D)	p-value, T-test
Y-maze memory (% Alternation)	54.4 (2.2)	56.7 (14.9)	0.81	63.4 (6.3)	62.2 (10.8)	0.85
	Sham Median (IQR)	ECS Median (IQR)	p-value, Mann-Whitney U-test	Sham Median (IQR)	ECS Median (IQR)	p-value, Mann-Whitney U-test
Exploratory behavior (% time in open arms)	35 (23-54)	7 (5-14)	0.11	49 (42-57)	17 (7-42)	0.23

Supplementary Table S3. S.D= standard deviation. IQR= interquartile range.

Table S4. Hippocampus 24 hours after treatment

	Females			Males		
	Sham Mean (S.D)	ECS Mean (S.D)	p-value, T-test	Sham Mean (S.D)	ECS Mean (S.D)	p-value, T-test
6E10 (plaque nr)	6.8 (5.0)	3.1 (3.4)	0.28	3.3 (3.1)	8.0 (6.6)	0.15
Amyloid Tetrameres (fold of β-actin)	Not available	Not available	Not available	0.90 (0.46)	0.53 (0.18)	0.20
Amyloid Oligomeres (fold of β-actin)	Not available	Not available	Not available	1.19 (0.88)	0.47 (0.22)	0.16
Iba1+ microglia (nr)	150.8 (22.3)	140.6 (9.6)	0.44	137.4 (15.1)	120.3 (26.3)	0.21

Supplementary Table S4. S.D= standard deviation

Table S5. Cortex 24 hours after treatment

	Females			Males		
	Sham Mean (S.D)	ECS Mean (S.D)	p-value, T-test	Sham Mean (S.D)	ECS Mean (S.D)	p-value, T-test
6E10 (plaque nr)	4.6 (3.2)	3.3 (5.3)	0.67	1.1 (1.2)	2.4 (2.7)	0.30
Iba1+ microglia (nr)	79.4 (9.2)	80.1 (7.8)	0.91	67.2 (10.6)	67.7 (12.2)	0.94

Supplementary Table S5. S.D= standard deviation

Supplementary Methods

Quantification of neurogenesis

Hippocampal neurogenesis is a robust marker of ECS treatment efficiency [1]. Therefore, we controlled for ECS treatment efficiency through staining for doublecortin (DCX) in hippocampus in free-floating sections as previously described [2]. Sections were stained with primary antibody anti-Doublecortin (1:2000, rabbit, Abcam) and secondary antibody anti-rabbit (1:1000, wavelength 488, Alexa Fluor, Life Technologies). DCX+ cells in DG were quantified using a fluorescence microscope (Olympus BX43, LRI, SE) (Nikon Eclipse 80i).

Quantification of fibrillary plaques

Amyloid plaques were stained with 0.5% thioflavin S. Sections were incubated during 5 min, rinsed for 3*10 min in PBS and mounted using ProLong Diamond Antifade mountant (Invitrogen). The thioflavinS-positive plaques were counted within regions of interest; dentate gyrus, CA2 and CA3 in hippocampus and cortical layer 4 and 5. Fibrillary plaque density was quantified as the number of thioflavin-S positive Aβ plaques/μm^2 in the cortex and hippocampus of sham and ECS mice, as described previously [2]. Images were taken with a Nikon confocal A1RHD laser-scanning microscope using a 10X objective. Analyses of the fluorescent labelled structures were done offline using Fiji ImageJ software (W. Rasband, National Institutes of Health). 3 brain sections/region/animal were analyzed (Sham: n= 10; ECS: n= 9).

Open field test

An open field test was used to control for baseline locomotion prior to the ECS treatment to assure that mice in different groups did not differ in baseline locomotor behavior. The mice were put in an empty arena (45x45 cm) and allowed to freely explore it for 10 minutes. An automated SMART system (Panlab, Barcelona, Spain) was used to measure the velocity of movements, distance moved and time spent in the center and periphery of the box. The box was cleaned with ethanol followed by water before each mouse was introduced to the arena.

Clasping scoring

Hindlimb clasping is a sign of disease progression and motor dysfunction, well known to increase over time in the 5xFAD model [3]. Throughout the study, hindlimb clasping behavior was assessed in the 5w group at 3 different time points (0, 5 and 8 weeks following the first ECS session) as described previously [4]. The mice were held in their tail and allowed to hang freely for 30 seconds while the clasping behavior was recorded and scored. Clasping was scored using a scale between 0 and 3, where 0 represented no clasping (normal), 1 represented initial signs of clasping or only clasping of one hind leg for at least 50% of the time, 2 represented clasping of both hind legs for at least 50% of the time, and 3 represented clasping of both hind legs for nearly 100% of the time.

References

1 Rotheneichner, P. *et al.* Hippocampal neurogenesis and antidepressive therapy: shocking relations. *Neural Plast* **2014**, 723915, doi:10.1155/2014/723915 (2014).

2 Boza-Serrano, A., Yang, Y., Paulus, A. & Deierborg, T. Innate immune alterations are elicited in microglial cells before plaque deposition in the Alzheimer's disease mouse model 5xFAD. *Sci Rep* **8**, 1550, doi:10.1038/s41598-018-19699-y (2018).

3 O'Leary, T. P., Robertson, A., Chipman, P. H., Rafuse, V. F. & Brown, R. E. Motor function deficits in the 12 month-old female 5xFAD mouse model of Alzheimer's disease. *Behav Brain Res* **337**, 256-263, doi:10.1016/j.bbr.2017.09.009 (2018).
4 Guyenet, S. J. *et al.* A simple composite phenotype scoring system for evaluating mouse models of cerebellar ataxia. *J Vis Exp*, doi:10.3791/1787 (2010).
